# Activation of MC1R with BMS-470539 attenuates neuroinflammation via cAMP/PKA/Nurr1 pathway after neonatal hypoxic-ischemic brain injury in rats

**DOI:** 10.1186/s12974-021-02078-2

**Published:** 2021-01-19

**Authors:** Shufeng Yu, Desislava Met Doycheva, Marcin Gamdzyk, Yijun Yang, Cameron Lenahan, Gaigai Li, Dujuan Li, Lifei Lian, Jiping Tang, Jun Lu, John H. Zhang

**Affiliations:** 1 Department of Pediatrics, Affiliated Haikou Hospital of Xiangya Medical College, Central South University, Haikou, 570208 China; 2 Department of Physiology and Pharmacology, Basic Sciences, School of Medicine, Loma Linda University, Loma Linda, CA 92354 USA; 3 Department of Hepatobiliary Surgery, Affiliated Haikou Hospital of Xiangya Medical College, Central South University, Haikou, 570208 China; 4 Burrell College of Osteopathic Medicine, Las Cruces, NM 88003 USA; 5 Department of Neurology, Tongji Hospital, Tongji Medical College, Huazhong University of Science and Technology, Wuhan, 430030 China; 6 Department of Pathology, Henan Provincial People’s Hospital, Zhengzhou, 453003 China; 7 Department of Anesthesiology, Neurosurgery and Neurology, School of Medicine, Loma Linda University, Loma Linda, CA 92354 USA

**Keywords:** Melanocortin-1 receptor, BMS-470539, Nurr1, Neuroinflammation, Microglial polarization, Neonatal hypoxia-ischemia

## Abstract

**Background:**

Microglia-mediated neuroinflammation plays a crucial role in the pathogenesis of hypoxic-ischemic (HI)-induced brain injury. Activation of melanocortin-1 receptor (MC1R) has been shown to exert anti-inflammatory and neuroprotective effects in several neurological diseases. In the present study, we have explored the role of MC1R activation on neuroinflammation and the potential underlying mechanisms after neonatal hypoxic-ischemic brain injury in rats.

**Methods:**

A total of 169 post-natal day 10 unsexed rat pups were used. HI was induced by right common carotid artery ligation followed by 2.5 h of hypoxia. BMS-470539, a specific selective MC1R agonist, was administered intranasally at 1 h after HI induction. To elucidate the potential underlying mechanism, MC1R CRISPR KO plasmid or Nurr1 CRISPR KO plasmid was administered via intracerebroventricular injection at 48 h before HI induction. Percent brain infarct area, short- and long-term neurobehavioral tests, Nissl staining, immunofluorescence staining, and Western blot were conducted.

**Results:**

The expression levels of MC1R and Nurr1 increased over time post-HI. MC1R and Nurr1 were expressed on microglia at 48 h post-HI. Activation of MC1R with BMS-470539 significantly reduced the percent infarct area, brain atrophy, and inflammation, and improved short- and long-term neurological deficits at 48 h and 28 days post-HI. MC1R activation increased the expression of CD206 (a microglial M2 marker) and reduced the expression of MPO. Moreover, activation of MC1R with BMS-470539 significantly increased the expression levels of MC1R, cAMP, p-PKA, and Nurr1, while downregulating the expression of pro-inflammatory cytokines (TNFα, IL-6, and IL-1β) at 48 h post-HI. However, knockout of MC1R or Nurr1 by specific CRISPR reversed the neuroprotective effects of MC1R activation post-HI.

**Conclusions:**

Our study demonstrated that activation of MC1R with BMS-470539 attenuated neuroinflammation, and improved neurological deficits after neonatal hypoxic-ischemic brain injury in rats. Such anti-inflammatory and neuroprotective effects were mediated, at least in part, via the cAMP/PKA/Nurr1 signaling pathway. Therefore, MC1R activation might be a promising therapeutic target for infants with hypoxic-ischemic encephalopathy (HIE).

**Supplementary Information:**

The online version contains supplementary material available at 10.1186/s12974-021-02078-2.

## Background

Hypoxic-ischemic encephalopathy (HIE) is one of the most common causes of perinatal brain injury and mortality in infants, which causes by the lack of oxygen and blood supply to the brain [1]. HIE has tremendous detrimental effects on the developing brain and leads to life-long neurological sequelae [2–4]. Several factors related to neonatal ischemic brain injury have been proposed, such as oxidative stress, inflammation, and apoptosis [5–8]. HIE still accounts for 23% of all neonatal deaths worldwide [4]. Thus far, there is not a well-established clinical treatment that can reduce brain damage and its long-term sequelae following perinatal HIE [9–11]. Therefore, studies regarding the pathogenesis of HIE are necessary to better understand brain injury mechanisms, and explore new therapeutic interventions that will help improve life-long neurological sequelae.

Mounting evidence has demonstrated that inflammation is a critical mechanism of hypoxic-ischemic (HI)-induced brain injury [8, 12–16]. Microglia are key innate immune cells of the central nervous system [12, 17], and when activated by HI, they initiate a cascade of inflammatory reactions that lead to neuronal damage [15]. Furthermore, overactivation of inflammatory responses would contribute to HI-induced secondary brain injury, causing permanent neurological function deficits [8, 12]. Microglia, when activated, develop into classically activated (M1-like, pro-inflammatory) or alternatively activated (M2-like, anti-inflammatory) phenotypes, a process termed polarization [13, 18, 19]. Emerging evidence has reported that pro-inflammatory cytokines, such as IL-1β, IL-6, and TNFα, increased post-HI [20, 21]. Methods for promoting the conversion of microglia phenotype from pro-inflammatory M1 into anti-inflammatory M2 form might be beneficial for HI brain injury. Moreover, previous studies have shown that inhibiting neuroinflammation has a neuroprotective effect in HI brain injury [5, 8, 16].

The melanocortin-1 receptor (MC1R) is one of five G protein-coupled receptors that belong to the melanocortin receptor subtype family (termed MC1R to MC5R), which reportedly plays a critical role in UV resistance and anti-inflammatory signaling [22–24]. It has been demonstrated that MC1R was expressed on neurons, astrocytes, and microglia in the brain [25, 26]. Recent studies have shown that activation of MC1R attenuated brain injury by inhibiting neuroinflammation in a mice model of intracerebral hemorrhage (ICH) [26]. Activation of MC1R has also been demonstrated to exert anti-inflammatory and neuroprotective effects in both acute and chronic inflammatory disease models, such as allergic rhinitis, experimental colitis, multiple sclerosis, and experimental autoimmune encephalomyelitis [27–29]. The α-melanocyte-stimulating hormone (α-MSH) is an endogenous nonselective agonist ligand of MC1R, MC3R, MC4R, and MC5R [22, 30]. BMS-470539, a novel potent and specific selective agonist of MC1R, has been shown to exert anti-inflammatory effects in lung inflammation and subarachnoid hemorrhage (SAH) [25, 31]. Emerging evidence has reported that activation of MC1R with BMS-470539 inhibited leukocytic infiltration and migration [32–34]. However, the effects of MC1R activation in neonatal HI have never been explored before.

Nurr1 (NR4A2), an orphan nuclear receptor, has been shown to provide significant neuroprotection via inhibition of pro-inflammatory cytokines in Parkinson’s disease, ICH, and acute cerebral ischemic/reperfusion [35–37]. Nurr1 expression reportedly could be upregulated by the PKA signaling pathway [38–41]. The increased intracellular cAMP levels enhanced the activation of PKA [42–44]. Moreover, activation of MC1R led to the activation of adenylyl cyclase (AC) and stimulated cAMP production [22, 24]. Hence, it is likely that Nurr1 may be a critical downstream molecule that contributes to the MC1R-mediated anti-inflammatory effects. However, to date, the role of Nurr1 in neonatal HIE-induced neuroinflammation remains unestablished.

Based on the aforementioned evidence, we hypothesized that the activation of MC1R by BMS-470539 could attenuate neuroinflammation and improve neurological functions, in part via the cAMP/PKA/Nurr1 signaling pathway in a rat neonatal HI model.

## Material and methods

### Animals

All procedures and protocols for this study were approved by the Institutional Animal Care and Use Committee (IUCAC) of Loma Linda University. All animals were cared for, and all studies were conducted in accordance with the US Public Health Service Policy on Humane Care and Use of Laboratory Animals. All experiments carried out on animals comply with the ARRIVE guidelines and were in accordance with the National Institutes of Health (NIH) Guide for the Care and Use of Laboratory Animals. Sprague Dawley rat mothers, with litters of 10–12 pups (P5-6, unsexed pups), were purchased from Harlan Labs (Livermore, CA). These pups were housed with their mothers in a humidity- and temperature-controlled environment with a regular 12 h light and dark cycle (lights on 6 a.m.–6 p.m.), and were raised with arbitrary access to breast milk, food, and water. The rat pups were retained for experiments until they reached post-natal day 10 (P10), which allowed them enough time to adjust. A total of 169 P10 unsexed rat pups (weighing 14~20 g) were used for this study. Among the 169 rat pups, 5 were excluded from the study due to death during or after hypoxia. The mortality rate was 3% (5/169). To decrease experimental bias and achieve unbiased results, all animals were randomly assigned to groups generated by excel. All investigators conducting the neurological tests and molecular experiments were blinded. Appropriate control groups were included for each intervention group.

### Neonatal hypoxia-ischemia brain injury rat model

The HI model was created based on the well-established Rice-Vannucci model as previously described [45]. Briefly, neonatal unsexed Sprague Dawley rat pups (P10) were placed in a temperature-controlled chamber for induction of general anesthesia. The pups were exposed to 3% isoflurane in air and were maintained at 2.5% isoflurane for anesthesia during surgery. After induction of anesthesia, the neck was prepared and draped following standard sterile techniques. Following this, a small midline neck incision on the right anterior neck was made with a No. 11 blade surgical knife (approximately 3–5 mm in length). Using gentle blunt dissection, the right common carotid artery was isolated and gently separated from the surrounding structures. The carotid artery was double ligated with a 5.0 surgical silk, and the carotid artery was severed between the ligations. The skin was closed with sutures. All bleeding was controlled with gentle pressure, as needed. The surgery was performed aseptically and the total time taken per surgery was 5–9 min. After the surgical procedure was completed, the pups were allowed to recover from anesthesia on a 37 °C blanket for 1 h. Thereafter, the pups were placed in a 500 ml airtight jar partially submerged in a 37 °C water bath to maintain a constant thermal environment. A gas mixture of 8% oxygen and 92% nitrogen was delivered into the jars through inlet and outlet portals. The pups were exposed to this gas mixture for 150 min. The flow rate of the gas was 4.0 l/min for the first 75 min and 3.5 l/min for the final 75 min. Afterward, the pups were returned to their mothers and monitored daily. The temperature was controlled with a heating blanket and incubator throughout the surgical and postoperative period. For sham animals, the right common carotid artery was exposed without ligation, and the animals were not exposed to hypoxic conditions.

### Experimental design

The experiment was designed as follows.

#### Experiment 1

To evaluate the time course expression of α-MSH, MC1R, and Nurr1 in the sham group and each group post-HI, the rat pups were randomly divided into 7 groups (*n* = 4/group): sham, 6 h HI, 12 h HI, 24 h HI, 48 h HI, 72 h HI, and 7 days HI. The expression of MC3R and MC4R in the sham and HI-48 h groups (*n* = 4/group) were analyzed by Western blot. Brain samples of the right (ipsilateral) hemisphere were collected for Western blot analysis. Moreover, to determine α-MSH expression on different cell types, an additional 4 rat pups (*n* = 2/group) in the sham and HI-24 h groups were used for immunofluorescence staining of α-MSH with NeuN (a marker for neurons), Iba-1(a marker for microglia), and GFAP (a marker for astrocytes). To assess the expression of α-MSH in the sham, HI-24 h contralateral hemisphere, and HI-24 h ipsilateral hemisphere, the rat pups were randomly divided into 2 groups (*n* = 4/group): sham and HI-24 h. Brain samples of the ipsilateral hemisphere and contralateral hemisphere were collected for Western blot analysis.

#### Experiment 2

Experiment 2 aims to evaluate the neuroprotective effects of MC1R activation with BMS-470539 on short-term outcomes at 48 h post-HI. Based on the dose response effect of BMS-470539 in previous studies [25, 33], three different doses of BMS-470539 (50 μg/kg, 160 μg/kg, 500 μg/kg) (sc-362716A, Santa Cruz, USA) were chosen and tested. The rat pups were randomly divided into 5 groups (*n* = 6/group): sham, HI + vehicle (10 μl sterile saline), HI + BMS-470539 (50 μg/kg, 10 μl), HI + BMS-470539 (160 μg/kg, 10 μl), and HI + BMS-470539 (500 μg/kg, 10 μl). BMS-470539 or vehicle (sterile saline) was administered intranasally at 1 h post-HI. Infarct area, short-term neurobehavioral tests: negative geotaxis, and body weight were evaluated at 48 h post-HI.

#### Experiment 3

The colocalization of MC1R and Nurr1 on microglia was characterized at 48 h post-HI. The rat pups were randomly divided into 3 groups (*n* = 2/group): sham, HI + vehicle (sterile saline), and HI + BMS-470539 (optimal dose). The optimal dose of BMS-470539 was selected based on the short-term outcomes study, which was also used for the following long-term outcome and mechanism studies.

#### Experiment 4

To evaluate the neuroprotective effects of MC1R activation with BMS-470539 on long-term outcomes at 28 days post-HI, the rat pups were randomly divided into 3 groups (*n* = 8/group): sham, HI + vehicle (sterile saline), and HI + BMS-470539 (optimal dose). BMS-470539 (optimal dose) or vehicle (sterile saline) was administered intranasally at 1 h post-HI. Long-term neurobehavioral test: foot-fault, rotarod, and Morris water maze were conducted at 28 days post-HI, and then, the rats were sacrificed for Nissl staining to measure brain tissue loss.

#### Experiment 5

To explore the underlying mechanisms of MC1R activation-mediated neuroprotective effects, rat pups were randomly divided into 6 groups (*n* = 6/group): sham, HI + vehicle (sterile saline), HI + BMS-470539 (optimal dose), HI + BMS-470539 (optimal dose) + MC1R KO CRISPR, HI + BMS-470539 (optimal dose) + Nurr1 KO CRISPR, and HI + BMS-470539 (optimal dose) + control CRISPR. BMS-470539 (optimal dose) or vehicle (sterile saline) was injected intranasally 1 h post-HI, while MC1R CRISPR, Nurr1 CRISPR, and control CRISPR were administered via intracerebroventricular injection at 48 h before HI. Infarct area, short-term neurobehavioral tests, body weight, immunofluorescence staining, and Western blot were measured at 48 h post-HI.

### Drug administration

Intranasal drug administration was performed at 1 h post-HI as previously described [25, 46]. The animals were placed in a supine position under 2% isoflurane anesthesia. A total of 10 μl of BMS-470539 (50 μg/kg, 160 μg/kg, and 500 μg/kg, sc-362716A, Santa Cruz) or vehicle (sterile saline) were administered intranasally at 1 h post-HI within 10 min. Next, 2 μl of BMS-470539 or vehicle (sterile saline) per drop was given every 2 min in alternating nares. The rat pups were then kept on their backs for an additional 5 min to allow for drug absorption prior to being returned to their cage.

### Intracerebroventricular injection

The rat pups were anesthetized with isoflurane, and then placed in a prone position and fixed in a stereotactic frame. A burr hole was made in the skull, and the needle of 10 μl Hamilton syringe (Hamilton Company, USA) was inserted through it into the right lateral ventricle. For the mechanism experiment, we used an engineered form of CRISPR-associated (Cas9) protein system. Briefly, the CRISPR protein Cas9 is directed to genomic target sites by specific guide RNAs, where it functions as an endonuclease, and further inactivates or activates specific target genes [47]. The specific MC1R or Nurr1 CRISPR KO plasmid only targets MC1R or Nurr1 gene. In this system, MC1R or Nurr1 CRISPR was used to knock out MC1R and Nurr1 gene expression in the rat brain. As previously described, CRISPR KO plasmids did not induce inflammation [48, 49]. Forty-eight hours before HI induction, MC1R CRISPR KO plasmid (Santa Cruz Biotechnology, USA), Nurr1 CRISPR KO plasmid (Santa Cruz Biotechnology, USA), or control CRISPR plasmid (Santa Cruz Biotechnology, USA) was administered via intracerebroventricular injection at 1.5 mm posteriors, 1.5 mm lateral to the bregma, and 1.7 mm deep into the ipsilateral hemisphere. A total of 2 μl of CRISPR was slowly administered in each pup intracerebroventricularly at a rate of 0.3 μl/min. The needle was then left in place for an additional 10 min to prevent leakage, and then withdrawn slowly over 5 min. The burr hole was then sealed with bone wax immediately after removing the needle. Then, the incision of the skin was sutured. When the rat pups were awakened from anesthesia, they would be returned to their cages.

### Infarct area measurements

As previously described, 2,3,5-triphenyltetrazolium chloride monohydrate (TTC) (Sigma-Aldrich, USA) staining was used to evaluate the infarct area [6, 46]. TTC staining is a standard and reliable method used to show the infarct area in models of ischemic stroke [46, 50, 51]. Briefly, the rat pups were anesthetized with isoflurane and then perfused transcardially with 25 ml 4 °C phosphate-buffered saline (PBS) at 48 h post-HI. The brains were removed and sectioned into 2 mm brain slices. A total of 5 coronal brain slices were prepared. Then, the slices were immersed in 2% TTC solution for 5 min at room temperature. The slices were washed with PBS and stored in 10% formaldehyde solution overnight. The brain slices were digitally photographed. Non-infarcted area of ipsilateral hemispheres and the total area of contralateral hemispheres were traced and analyzed using ImageJ software (NIH, USA). The percent of infarct area for each slice was calculated using the following formula: [(total area of contralateral hemisphere) − (area of non-infarcted area of ipsilateral hemisphere)]/(total area of contralateral hemisphere × 2) × 100% [5, 6, 46]. The area was calculated for each slice, and the average area of each slice was taken to represent the percentage of infarcted area for that rat.

### Neurological evaluation

Neurobehavioral tests were conducted by two blinded investigators in an unbiased setup at either 48 h or 28 days post-HI, as previously reported [5, 6]. Negative geotaxis tests were conducted at 48 h post-HI for evaluating short-term neurological function. Foot-fault, rotarod, and Morris water maze were conducted at 28 days post-HI to evaluate long-term neurological function.

### Short-term neurological evaluation

To evaluate short-term neurological function, negative geotaxis test was conducted as previously described at 48 h post-HI [6]. In negative geotaxis, rat pups were placed head downward on an inclined board (45°), and the time it took for the rat pups to turn their bodies around and face upward of the board was recorded. The maximum testing time was 60 s [46].

### Long-term neurological evaluation

To evaluate long-term neurological function, foot-fault, rotarod, and Morris water maze tests were conducted at 28 days post-HI, as previously described [6, 46].

#### Foot-fault test

The rat pups were placed on a horizontal grid floor (square size 20 cm × 40 cm with a mesh size of 4 cm^2^), which was elevated 1 m above the floor for 1 min. The trial was recorded for post-test analysis. Foot-fault was defined when the pup could not place a forelimb or hindlimb accurately and the paw fell between the grid bars. The number of foot-faults for each rat was recorded by a video device and analyzed by an investigator blinded to the experimental groups.

#### Rotarod test

This test was used to assess motor impairment by using an accelerating rotarod (Columbus Instruments Rotamex, USA). The rat pups were placed on an accelerating rotarod, and the time it took for them to fall was recorded. The rotation speed started from 5 or 10 rpm separately with an acceleration of 2 rpm/5 s. The maximum testing time was 60 s, and the time taken more than 60 s was recorded as 60 s.

#### Morris water maze test

This test was used to evaluate the animal ability of learning and memory. Each rat performed 5 trials per day for 6 days. Between successive trials, there was a 10-min interval. All trials lasted no more than 60 s. Briefly, the rats were trained using a visible platform (diameter 10 cm, cued test, block 1) in a pool of water on day 1. If the rats had not discovered the platform in 60 s, they were manually guided to the platform. On days 2–5, the latency to find a platform submerged 1 cm below the water was measured (memory test, blocks 2–5). On day 6, the platform was removed, and the time spent in the platform quadrant was tested (probe trial, 1 min trial, block 6). A video recording system traced all of the animals’ activities. The animals’ swimming path was measured for the quantification of distance, latency, and swimming speed by the video tracking system SMART-2000 (San Diego Instruments Inc, USA).

### Western blotting analysis

Western blot was conducted as previously described [6, 46]. Brain tissues were used for Western blot analysis after first being stained with TTC and imaged. Previous studies demonstrated that TTC-stained brain tissues could be used for quantitative gene and protein expression analyses using real-time polymerase chain reaction (RT-PCR) and Western blot in models of ischemic stroke including in the HI rat model without altering the expression of proteins [52–54]. After TTC staining, slices were imaged and recorded at 48 h post-HI, and the brain slices were instantly divided into the ipsilateral and contralateral cerebrums, snap frozen in liquid nitrogen, and then stored in a − 80 °C freezer for Western blot. As previously reported, the ipsilateral hemisphere was used to measure the protein expression by Western blot in a rat model of HI brain injury [5–7, 46, 52]. To obtain whole-cell lysates, the right/ipsilateral hemisphere tissue was homogenized in RIPA lysis buffer (Santa Cruz Biotechnology, USA), and then centrifuged at 14,000*g* at 4 °C for 20 min. Then, the supernatant was collected, and protein concentration was determined using a detergent compatibility assay (Bio-Rad, Dc protein assay). After each sample of protein concentration was calculated using the spectrophotometer (ThermoFisher Scientific, USA), 40 μg of protein from each sample was loaded into 8–12% SDS-PAGE gel, and then electrophoresed. Then, the protein was transferred onto nitrocellulose membranes (0.45 μm), which was blocked with 5% non-fat blocking grade milk (Bio-Rad, USA) for 1 h at room temperature. The membranes were incubated with the respective primary antibody overnight at 4 °C [5, 6, 46]. The following primary antibodies were used: MC1R (1:500, PA5-75342, ThermoFisher, USA), MC3R (1:1000, PA5-88171, ThermoFisher, USA), anti-MC4R (1:500, ab24233, Abcam, USA), α-MSH (1:1000, orb229289, biorbyt, USA), anti-Nurr1 (1:500, ab41917, Abcam, USA), anti-cAMP (1:1000, ab76238, Abcam, USA), p-PKA (1:1000, #4781S, Cell Signaling, USA), PKA (1:1000, #4782S, Cell Signaling, USA), anti-IL-1β (1:500, ab9722, Abcam, USA), anti-IL-6 (1:1000, ab9324, Abcam, USA), anti-TNFα (1:1000, ab6671, Abcam, USA), and anti-β-actin (1: 3000, sc-47778, Santa Cruz, USA). The following day, the membranes were incubated with secondary antibodies (1:2000, sc-2357, Santa Cruz, USA) for 2 h at room temperature. Immunoblots were then visualized with ECL Plus chemiluminescence reagent kit (Amersham Bioscience, Arlington Heights, IL), and then analyzed using ImageJ (NIH, USA). The results were displayed as relative density (grayscale value of the target proteins/β-actin or total proteins).

### Immunofluorescence staining

The rat pups were anesthetized at 24 h or 48 h post-HI, and then perfused transcardially with ice-cold PBS and 10% formalin. The brains were removed and fixed with 10% formalin overnight. Then, the brains were immersed in a 20% sucrose solution for 2 days, then transferred into 30% sucrose solution for 2 days. Finally, the brains were embedded into OCT compound (Scigen Scientific, USA) and frozen at − 80 °C. The frozen brains were cut into 10 μm-thick coronal sections at − 20 °C with a cryostat (CM3050S-3, Leica Microsystems, USA). The sectioned tissue was then fixed on glass slides and used for immunofluorescence staining which was performed as described previously [5, 6]. The brain slices were washed with PBS three times for 5–10 min, then incubated in 0.3% Triton X-100 for 15 min at room temperature. Then, the brain slices were washed with PBS three times for 5 min each, and blocked with 5% donkey serum at room temperature for an hour. The sections were incubated at 4 °C overnight with the following primary antibodies: MC1R (1:50, PA5-75342, ThermoFisher, USA), anti-α-MSH (1:50, ab123811, Abcam, USA), anti-Nurr1 (1:50, ab41917, Abcam, USA), anti-IL-1β (1:100, ab9722, Abcam, USA), anti-Myeloperoxidase (MPO) (1:100, ab65871, Abcam, USA), anti-Mannose Receptor (CD206) (1:100, ab64693, Abcam, USA), anti-CD11b/c (1:100, ab1211, Abcam, USA), anti-Iba1 (1:100, ab5076, Abcam, USA), anti-GFAP (1:100, ab53554, Abcam, USA), and anti-NeuN (1:100, ab104224, Abcam, USA). After washing with PBS, secondary antibodies were applied at a dilution of 1:200 for 2 h at room temperature. Slides were washed with PBS three times for 10 min. Finally, sections were covered with DAPI (Vector Laboratories Inc, USA), and slides were visualized with a fluorescence microscope Leica DMi8 (Leica Microsystems, Germany) and analyzed by Leica Application Suite software. To evaluate the numbers of α-MSH^+^, CD206^+^ CD11 b/c^+^, MPO^+^, and IL-1β^+^ cells, one field from each of the three different sections per brain was averaged and expressed as positive cells per square millimeter.

### Nissl staining and brain tissue loss

Nissl staining was conducted according to a previous report [6]. Briefly, the rats were treated as described above for immunofluorescence staining. The frozen brains were cut into 20 μm-thick coronal sections at − 20 °C with a cryostat (CM3050S-3, Leica Microsystems, USA). The sections were dehydrated in 95% and 70% ethanol for 1 min each, rinsed in tap water, and then distilled water for 30 s. Next, the sections were stained with 0.5% Cresyl Violet (Sigma-Aldrich, USA) for 3 min, and then washed in distilled water for 10 s and 30 s. The sections were then dehydrated in 100% ethanol and xylene two times for 1.5 min each, and covered with DPX (Sigma-Aldrich, USA). The sections were imaged using a microscope (Olympus-BX51) equipped with MagnaFire SP 2.1B software (Olympus). Each of the three sections per brain was averaged and then measured with ImageJ. The percentage of brain tissue loss = (contralateral hemisphere − ipsilateral hemisphere)/contralateral hemisphere × 100% [46].

### Statistical analysis

All data were presented as the mean and standard deviation (mean ± SD). Statistical analysis was performed using GraphPad Prism 7 (Graph Pad Software, San Diego, USA). The appropriate parametric test was used (one-way ANOVA or Student’s *t* test) followed by Tukey’s post hoc test or Student-Newman-Keuls test if necessary. The data of long-term neurological functions were analyzed using two-way ANOVA, A *p* value < 0.05 was considered statistically significant.

## Results

### Expression levels of endogenous α-MSH, MC1R, and Nurr1 were increased post-HI

**Fig. 1:**
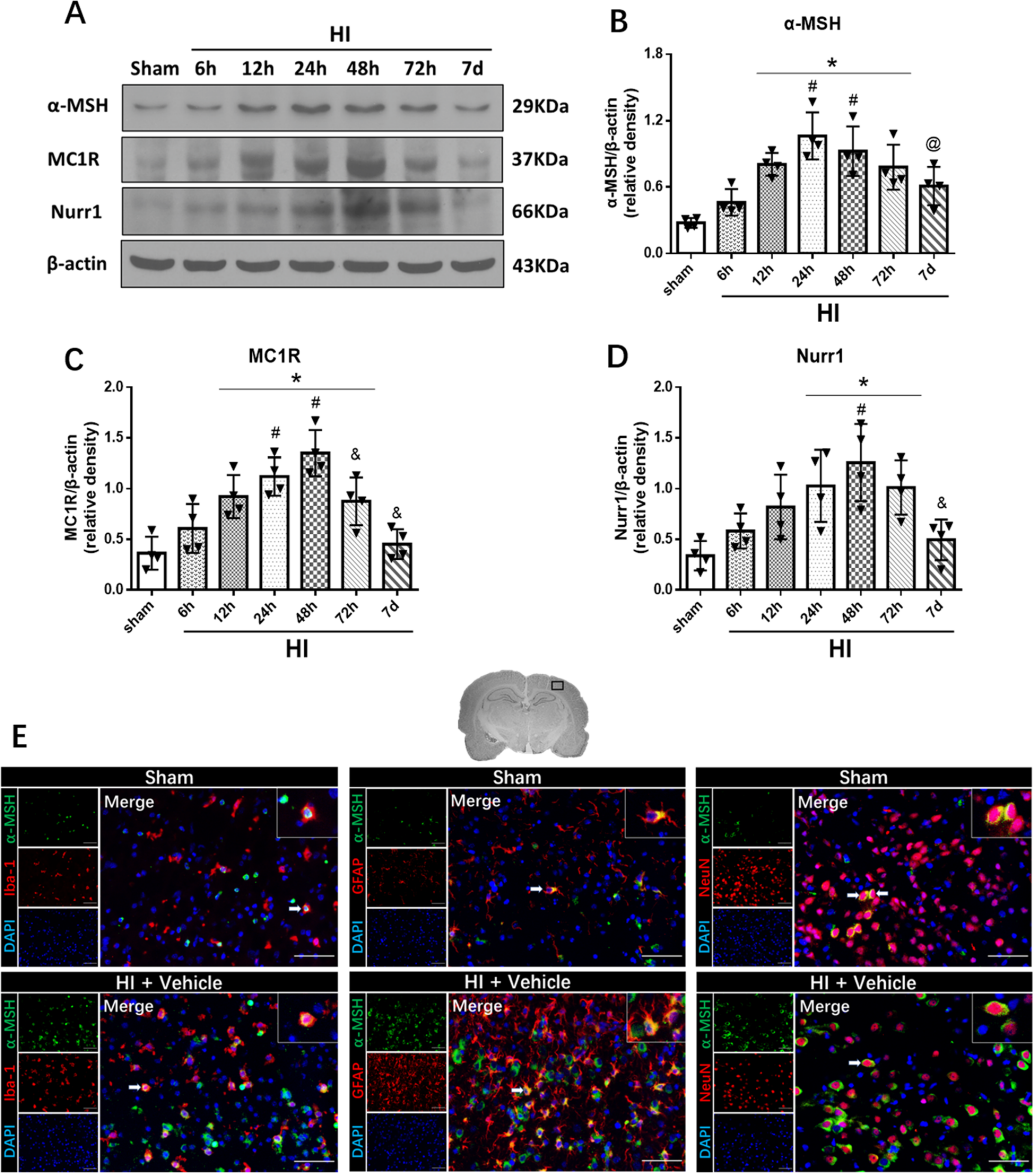
Expression levels of α-MSH, MC1R, and Nurr1 post-HI. **a** Representative pictures of Western blot data. **b** Western blot data showed that the expression levels of α-MSH significantly increased at 12 h post-HI, and peaked at 24 h. **c**, **d** The endogenous expression levels of MC1R and Nurr1 increased over time and peaked at 48 h post-HI. Data were represented as mean ± SD. Statistical differences between groups were analyzed using one-way ANOVA, followed by Tukey’s post hoc test (*p < 0.05 versus sham, ^#^*p* < 0.05 versus 6 h HI, ^@^*p* < 0.05 versus 24 h HI, ^&^*p* < 0.05 versus 48 h HI; *n* = 4 per group). **e** Representative microphotographs of double immunofluorescence of α-MSH (green) with microglia (Iba-1, red), astrocyte (GFAP, red), and neuron (NeuN, red) in the peri-infarcted area at 24 h post-HI. DAPI was stained blue. Merged images showed that α-MSH was colocalized with microglia, astrocytes, and neurons. *n* = 2 per group. Scale bar = 100 μm

The endogenous expression levels of α-MSH, MC1R, and Nurr1, in the ipsilateral hemispheric brain, were measured at 6 h, 12 h, 24 h, 48 h, 72 h, and 7 days post-HI. Figure 1a shows representative Western blot bands of the endogenous expression levels of α-MSH, MC1R, and Nurr1. The expression levels of α-MSH started to significantly increase from 12 h, and peaking at 24 h post-HI compared to the sham group (*p* < 0.05, Fig. 1a, b). The expression level of α-MSH significantly increased in the ipsilateral hemisphere compared to the sham group and the contralateral hemisphere at 24 h post-HI (*p* < 0.05, Additional file 1: Figure S1). However, there is no significant difference between the sham group and the contralateral hemisphere at 24 h post-HI (*p* > 0.05, Additional file 1: Figure S1). MC1R and Nurr1 expression levels increased over time, and reached the highest point at 48 h, and then returned to base levels by 7 days post-HI (*p* < 0.05, Fig. 1a, c, d). MC3R and MC4R expression levels significantly increased at 48 h post-HI compared to the sham group (*p* < 0.05, Additional file 1: Figure S2).

Double immunofluorescence staining of α-MSH with Iba-1, GFAP, and NeuN was performed in the sham group and 24 h vehicle group post-HI (Fig. 1e). The results showed that α-MSH was expressed on microglia, astrocytes, and neurons. Moreover, α-MSH tended to increase in microglia, neurons, and astrocytes after HI. And the expression of α-MSH on neurons was higher compared to microglia and astrocytes (*p* < 0.05, Fig. 1e, Additional file 1: Figure S3).

### Activation of MC1R with BMS-470539 reduced infarct area, also improved body weight and short-term neurological function at 48 h post-HI

**Fig. 2:**
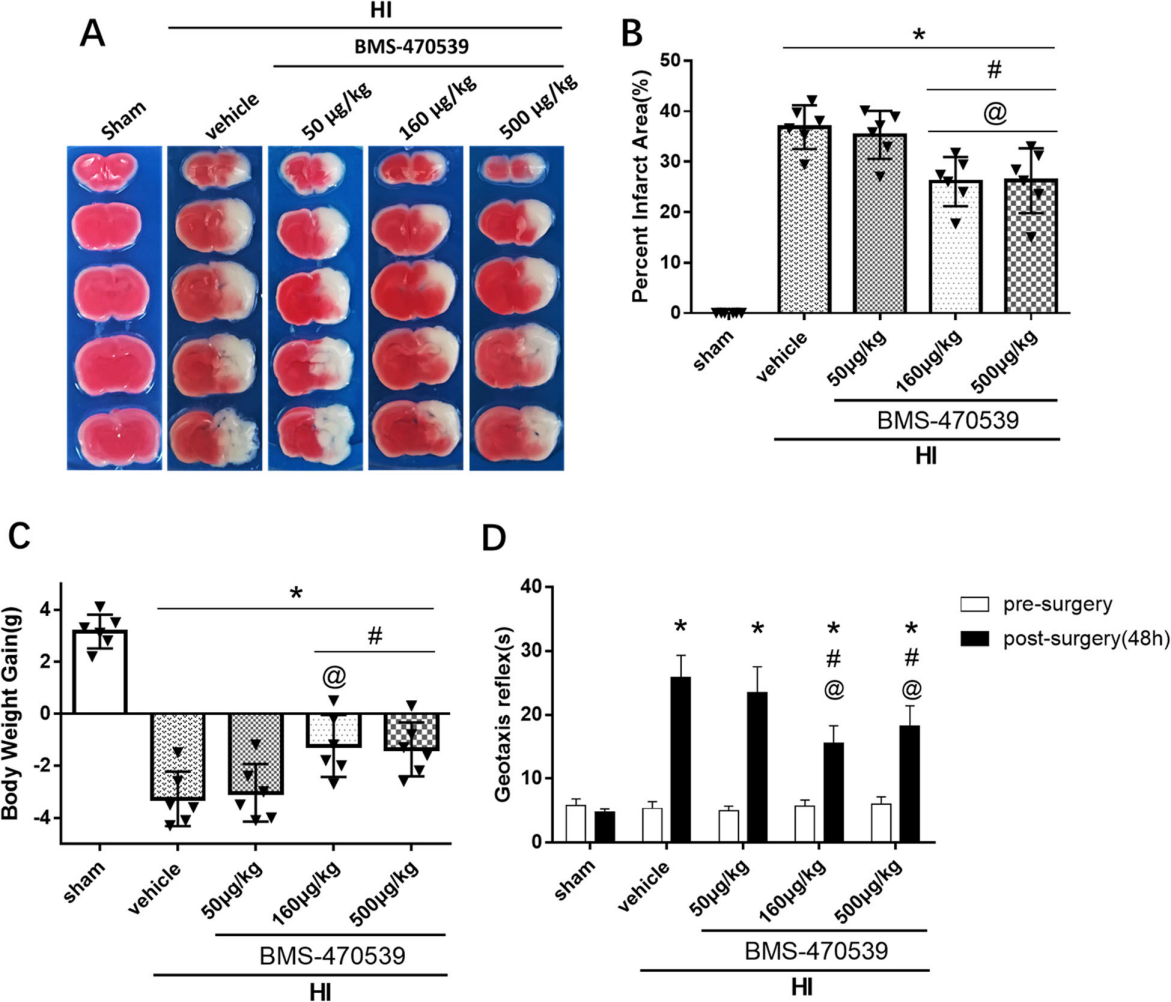
Effect of MC1R activation on brain infarct area, body weight, and short-term neurological function at 48 h post-HI. **a**, **b** TTC staining showed that medium (160 μg/kg) and high (500 μg/kg) doses of BMS-470539 treatment significantly reduced brain infarct area compared to the vehicle. **c** Rats in the vehicle group showed to lose significant weight compared to the sham group, medium (160 μg/kg) and high (500 μg/kg) doses of BMS-470539 treatment groups. **d** Geotaxis reflex showed that medium (160 μg/kg) and high (500 μg/kg) doses of BMS-470539 significantly improved neurological function compared to the vehicle group. Data were represented as mean ± SD. *p < 0.05 versus sham, ^#^*p* < 0.05 versus vehicle, ^@^*p* < 0.05 versus BMS-470539 (50 μg/kg). *n* = 6 per group

To determine the optimal dose of BMS-470539 needed to reduce the brain infarct area, three doses were used: low (50 μg/kg), medium (160 μg/kg), and high (500 μg/kg). TTC staining results showed that the medium (160 μg/kg) and high (500 μg/kg) doses of BMS-470539 treatment significantly reduced infarct area at 48 h post-HI when compared with the vehicle group (*p* < 0.05, Fig. 2a, b). No significant difference in the infarcted area was found in the low dose of BMS-470539 (50 μg/kg) group when compared with the vehicle group (*p* > 0.05, Fig. 2a, b). The rat pups in the vehicle group showed to lost significant body weight compared with the sham, medium (160 μg/kg), and high (500 μg/kg) doses of BMS-470539 treatment groups at 48 h post-HI (*p* < 0.05, Fig. 2c). However, no significant difference in body weight loss was observed between medium (160 μg/kg) and high (500 μg/kg) doses of BMS-470539 groups (*p* > 0.05, Fig. 2c). Short-term neurological function was evaluated using geotaxis test. Geotaxis test was performed on the rat pups, and the test results showed that pups performed significantly worse at 48 h post-HI compared to the sham group (*p* < 0.05, Fig. 2d). Both medium and high doses of BMS-470539 significantly improved short-term neurological function compared to the vehicle group and the low-dose BMS-470539 group (*p* < 0.05, Fig. 2d). However, there is no significant difference between these groups (*p* < 0.05, Fig. 2d). Based on the above results and principle of priority, we chose the medium dose of BMS-470539 for long-term outcome and mechanism studies.

### Immunofluorescence staining for MC1R and Nurr1 expression, as well as colocalization with microglia at 48 h post-HI

**Fig. 3:**
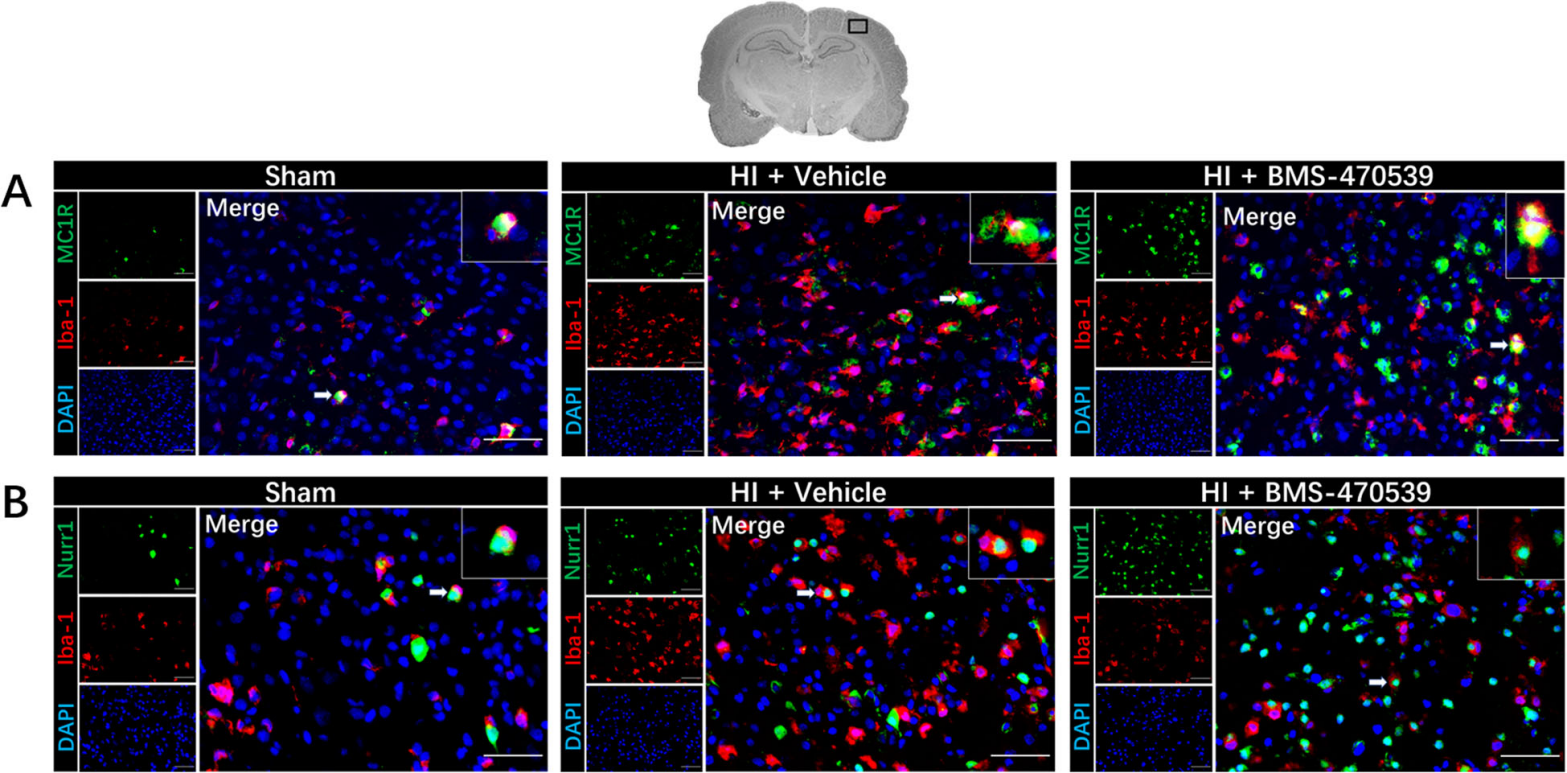
Immunofluorescence staining of MC1R and Nurr1 with microglia in the brain at 48 h post-HI. Immunofluorescence staining showed an increase expression of MC1R (**a**) and Nurr1 (**b**) on microglia in the vehicle group when compared with the sham group, and further increased expression of MC1R (**a**) and Nurr1 (**b**) in the BMS-470359 treatment group. Merge showed the colocalization of MC1R and Nurr1 on microglia. Microglia were stained red. MC1R and Nurr1 were stained green. DAPI was stained blue. *n* = 2 per group. Scale bar = 100 μm

Immunofluorescence staining showed that MC1R and Nurr1 were increased post-HI and BMS-470359 treatment group (Fig. 3). Furthermore, the expression of MC1R and Nurr1 on microglia were higher in the BMS-470359 treatment group compared to the vehicle group (Fig. 3). MC1R and Nurr1 showed colocalization with microglia in the sham, vehicle, and treatment groups (Fig. 3).

### Activation of MC1R with BMS-470539 reduced brain atrophy and improved long-term neurological function at 28 days post-HI

**Fig. 4:**
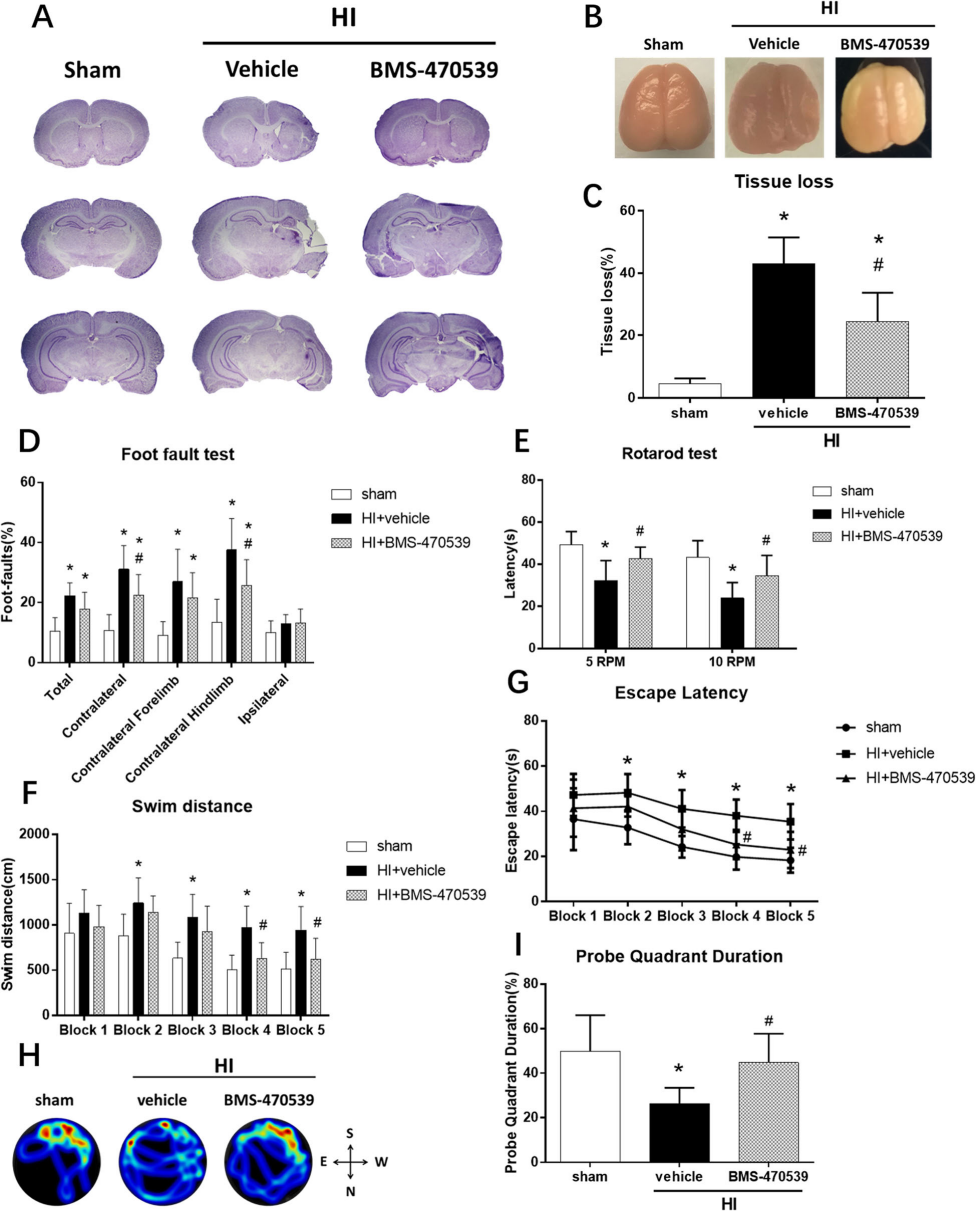
Effects of MC1R activation on brain atrophy and long-term neurological function at 28 days post-HI. **a**–**c** Representative pictures of Nissl staining the brain slices showing tissue loss in the ipsilateral hemisphere. The BMS-470539 treatment group significantly reduced the percentage of tissue loss and brain atrophy compared to the vehicle group. **d**, **e** Activation of MC1R with BMS-470539 significantly improved sensorimotor function as shown by foot-fault and rotarod tests. **f**–**i** The BMS-470539 treatment group significantly improved in spatial memory and learning abilities compared to the vehicle group, as demonstrated by less swim distance to find the platform (**f**), less escape latency (**g**), and more time spent in the target quadrant during the probe test (**h**, **i**). **h** Representative image of swim track in probe trial. Data was represented as mean ± SD. Statistical differences between groups were analyzed using one-way ANOVA or two-way ANOVA followed by Tukey multiple-comparison post hoc analysis. *p < 0.05 versus sham; ^#^*p* < 0.05 versus HI + vehicle; *n* = 8 per group

HI resulted in severe brain atrophy in the ipsilateral hemispheres at 28 days post-HI, while intranasal administration of BMS-470539 treatment significantly reduced brain atrophy compared to the vehicle group (Fig. 4a–c). Nissl staining showed that activation of MC1R with BMS-470539 significantly attenuated the percentage of brain tissue loss (*p* < 0.05, Fig. 4a, c).

To assess the effects of MC1R activation with BMS-470539 on the long-term neurological impairments induced by HI, neurological functions were evaluated by foot-fault, rotarod, and Morris water maze test at 28 days post-HI. In all these tests, rats in the vehicle group performed worse compared to the sham and BMS-470539 treatment groups. Activation of MC1R with BMS-470539 significantly improved sensorimotor function in foot-fault and rotarod tests. In the foot-fault test, animals in the vehicle group showed more total foot-faults, as well as more foot-faults in the contralateral (left) side than the sham group. However, activation of MC1R with BMS-470539 significantly reduced the percentage of foot-faults in the contralateral side and contralateral hindlimbs compared to the vehicle group at 28 days post-HI (*p* < 0.05, Fig. 4d). In the rotarod test, the animals in the vehicle group had a significantly shorter falling latency compared to the sham group. However, activation of MC1R with BMS-470539 significantly improved the sensorimotor function by increasing the falling latency at both the 5 rpm and 10 rpm acceleration when compared with the vehicle group at 28 days post-HI (*p* < 0.05, Fig. 4e). In the Morris water maze test, the animals in the vehicle group showed a significant reduction in spatial memory and learning abilities compared to the sham group (*p* < 0.05, Fig. 4f–i). However, activation of MC1R with BMS-470539 significantly improved in spatial memory and learning abilities compared to the vehicle group. This was demonstrated by a shorter swimming distance to locate the platform (*p* < 0.05, Fig. 4f), a shorter time to find the platform (*p* < 0.05, Fig. 4g), and more time spent in the target quadrant during the probe test (*p* < 0.05, Fig. 4h, i).

### In vivo knockout of MC1R and Nurr1 reversed the neuroprotective effects of BMS-470539 at 48 h post-HI

**Fig. 5:**
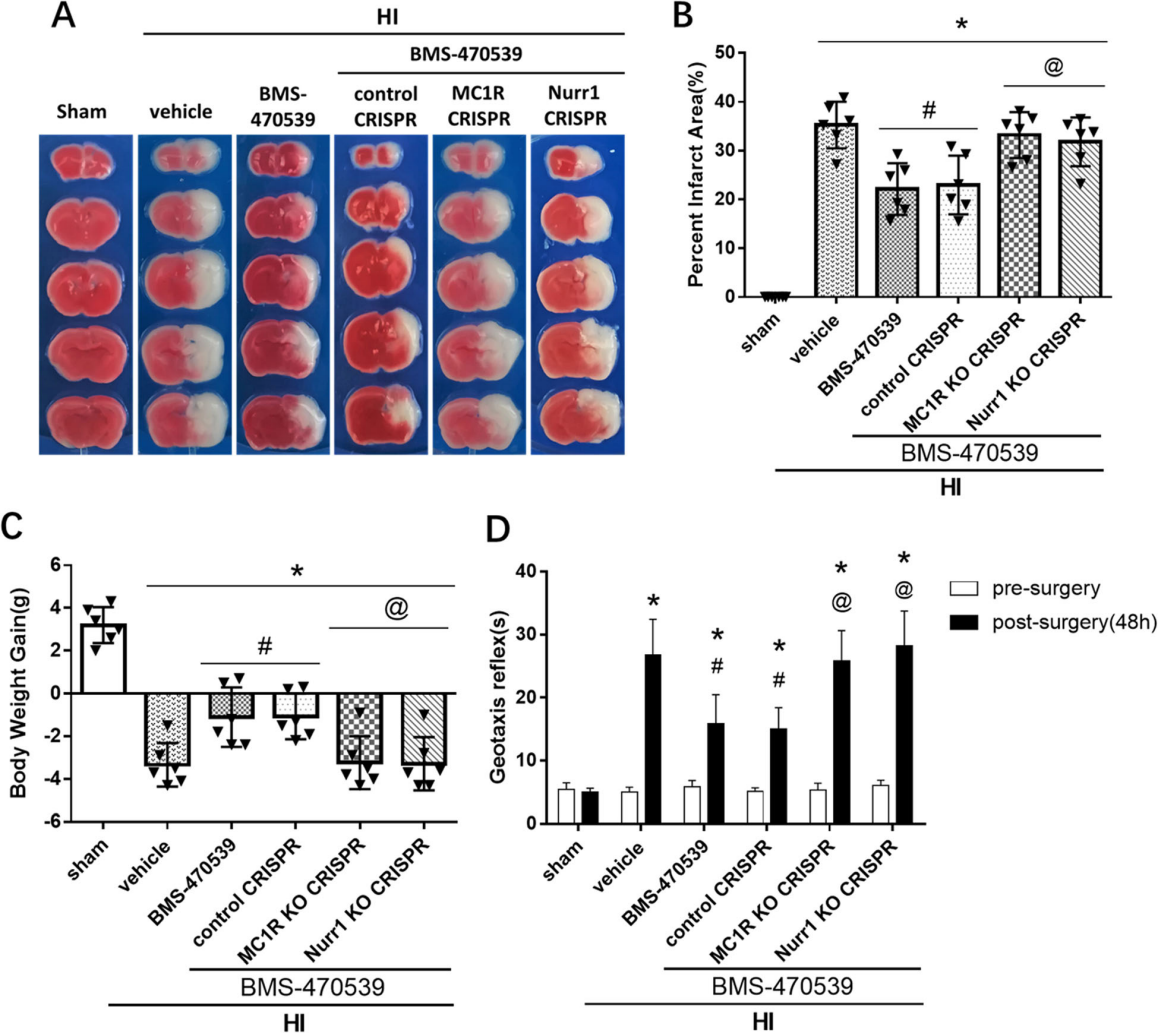
Effects of knockout MC1R and Nurr1 on infarct area, body weight, and neurological function at 48 h post-HI. **a**, **b** The infarct area was significantly increased in both treatment groups with CRISPR compared to the HI + BMS-470539 group or HI + BMS-470539 + control CRISPR group. **c** Activation of MC1R with BMS-470539 significantly reduced body weight loss compared to the vehicle. However, both treatment groups with CRISPR interventions significantly reversed these effects compared to the HI + BMS-470539 group or HI + BMS-470539 + control CRISPR group. **d** The geotaxis test showed that the rat pups treated with BMS-470539 and either MC1R CRISPR or Nurr1 CRISPR had significantly impaired neurological function compared to the corresponding controls. Data was represented as mean ± SD. *p < 0.05 versus sham, ^#^*p* < 0.05 versus HI + vehicle, ^@^*p* < 0.05 HI + BMS-470539 or HI + BMS-470539 + control CRISPR. *n* = 6 per group

To assess whether pathway interventions can affect neuroprotection and increase brain infarct area, MC1R CRISPR and Nurr1 CRISPR were used to knock out MC1R and Nurr1 gene expression in the rat brain. TTC staining data showed that knockout of MC1R or Nurr1 by CRISPR significantly abolished the neuroprotective effects of BMS-470539, which was seen as the significant increase in the percentage of infarct area as compared to the HI + BMS-470539 treatment group or HI + BMS-470539 + control CRISPR group (*p* < 0.05, Fig. 5a, b). There was a significant change in the body weight of rat pups in the group treated with BMS-470539 and either MC1R CRISPR or Nurr1 CRISPR compared to the HI + BMS-470539 treatment group or HI + BMS-470539 + control CRISPR group (*p* < 0.05, Fig. 5c). Furthermore, the geotaxis test revealed that the rat pups administered with BMS-470539 treatment and then with MC1R CRISPR or Nurr1 CRISPR had significantly impaired neurological function compared to the corresponding controls (*p* < 0.05, Fig. 5d).

### Activation of MC1R with BMS-470539 promoted microglial M2 polarization at 48 h post-HI.

**Fig. 6:**
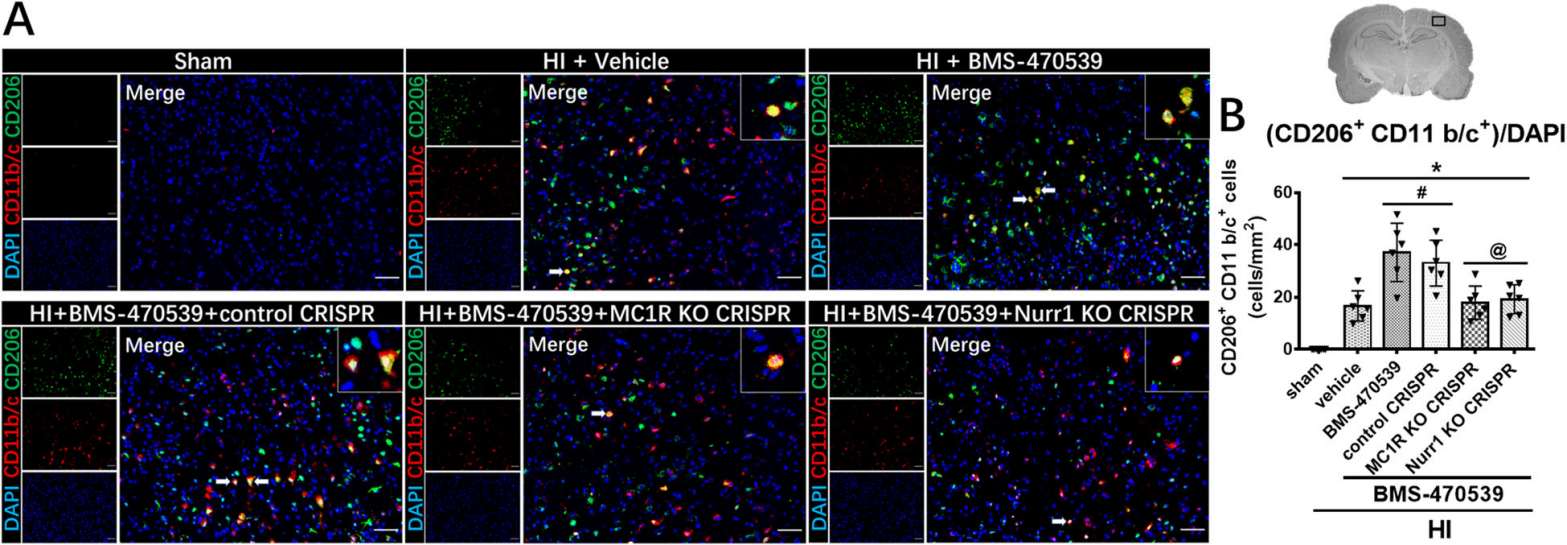
MC1R activation increased the expression of CD206 at 48 h post-HI. Representative microphotographs of immunofluorescence staining and quantification of CD206 with microglia (CD11 b/c) at 48 h post-HI. **a**, **b** The number of CD206^+^ CD11 b/c^+^ cells significantly increased in the vehicle group compared to the sham group. Activation of MC1R with BMS-470539 further increased the number of CD206^+^ CD11 b/c^+^ cells compared to the vehicle group, while knockout CRISPR interventions significantly reversed these effects. CD206 was green. CD11 b/c was red. Blue was for DAPI. Scale bar = 100 μm. Data was represented as mean ± SD. The one-way ANOVA was followed by Tukey’s post hoc test (*p < 0.05 versus sham; ^#^*p* < 0.05 versus HI + vehicle; ^@^*p* < 0.05 HI + BMS-470539 or HI + BMS-470539 + control CRISPR; *n* = 6 per group)

Immunofluorescence staining of CD206 with microglia (CD11 b/c) was performed to evaluate microglial M2 polarization. The results of immunofluorescence staining data showed that the number of CD206^+^ CD11 b/c^+^ cells significantly increased in the vehicle group compared to the sham group at 48 h post-HI (*p* < 0.05, Fig. 6a, b). Moreover, activation of MC1R with BMS-470539 further increased the number of CD206^+^ CD11 b/c^+^ cells compared to the vehicle group, while both treatment groups with CRISPR interventions significantly reversed these effects (*p* < 0.05, Fig. 6a, b).

### Activation of MC1R with BMS-470539 suppressed neuroinflammation, and reduced peripheral immune cell infiltration at 48 h post-HI

**Fig. 7:**
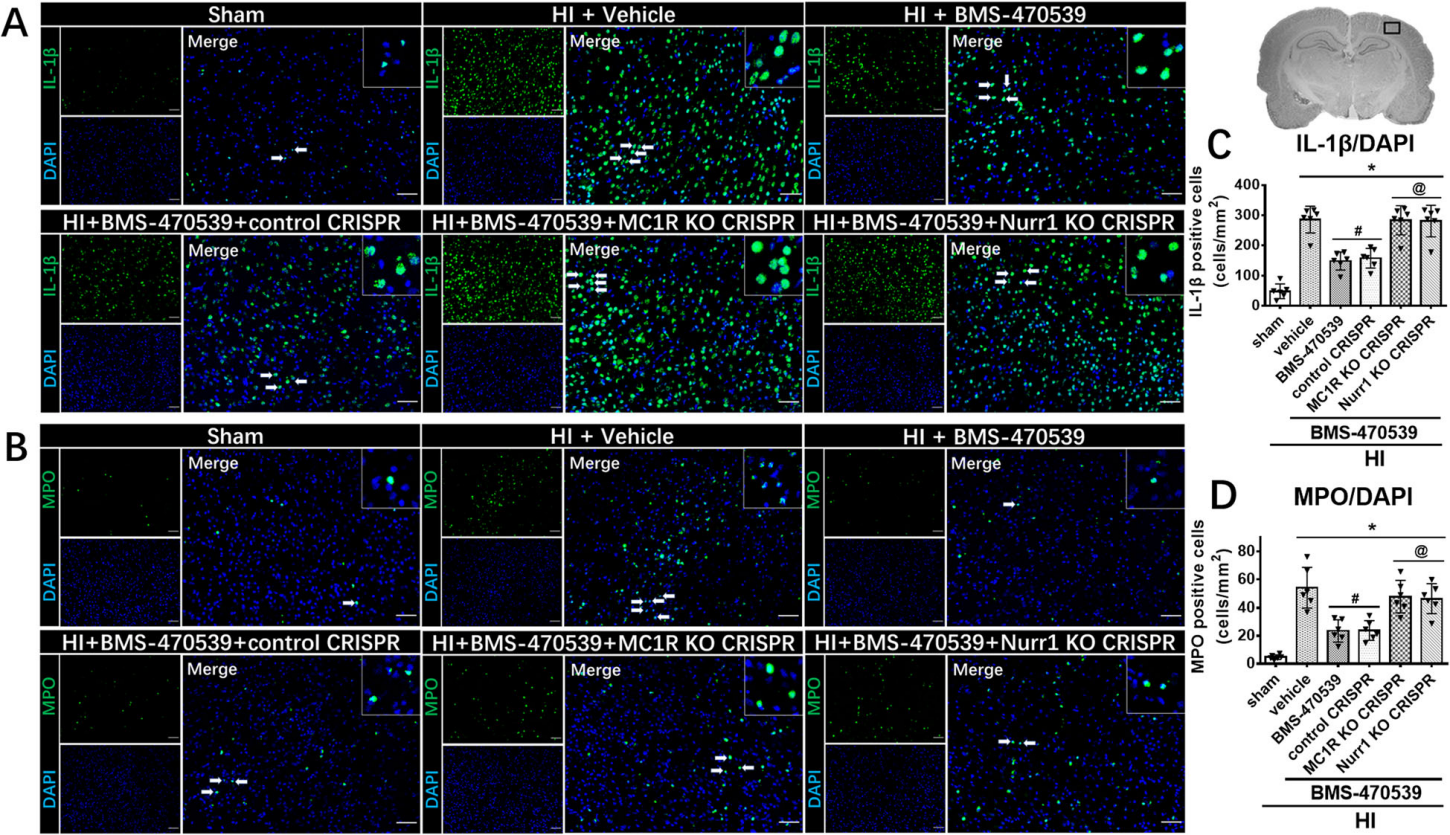
MC1R activation on immunofluorescence staining of IL-1β and MPO at 48 h post-HI. Representative microphotographs of immunofluorescence staining and quantification of **a**, **c** IL-1β-positive cells and **b**, **d** MPO-positive cells. The number of IL-1β-positive cells or MPO-positive cells was significantly increased in the vehicle group compared to the sham group. BMS-470539 treatment significantly suppressed neuroinflammation, while these effects were reversed by knockout of MC1R or Nurr1 with CRISPR. IL-1β and MPO were green. Blue was for DAPI. Scale bar = 100 μm. Data was represented as mean ± SD. The one-way ANOVA was followed by Tukey’s post hoc test (*p < 0.05 versus sham; ^#^*p* < 0.05 versus HI + vehicle; ^@^*p* < 0.05 HI + BMS-470539 or HI + BMS-470539 + control CRISPR; *n* = 6 per group)

Immunofluorescence staining of MPO and IL-1β were performed to evaluate the neuroinflammation. Compared to the sham group, the number of IL-1β- and MPO-positive cells was significantly increased in the vehicle group at 48 h post-HI (*p* < 0.05, Fig. 7a, c and b, d). Activation of MC1R with BMS-470539 significantly decreased the number of IL-1β- and MPO-positive cells compared to the vehicle group, while knockout CRISPR interventions significantly abolished the effects of MC1R activation. (*p* < 0.05, Fig. 7a, c and b, d).

### Activation of MC1R with BMS-470539 downregulated expression of pro-inflammatory cytokines through the cAMP/PKA/Nurr1 signaling pathway at 48 h post-HI

**Fig. 8:**
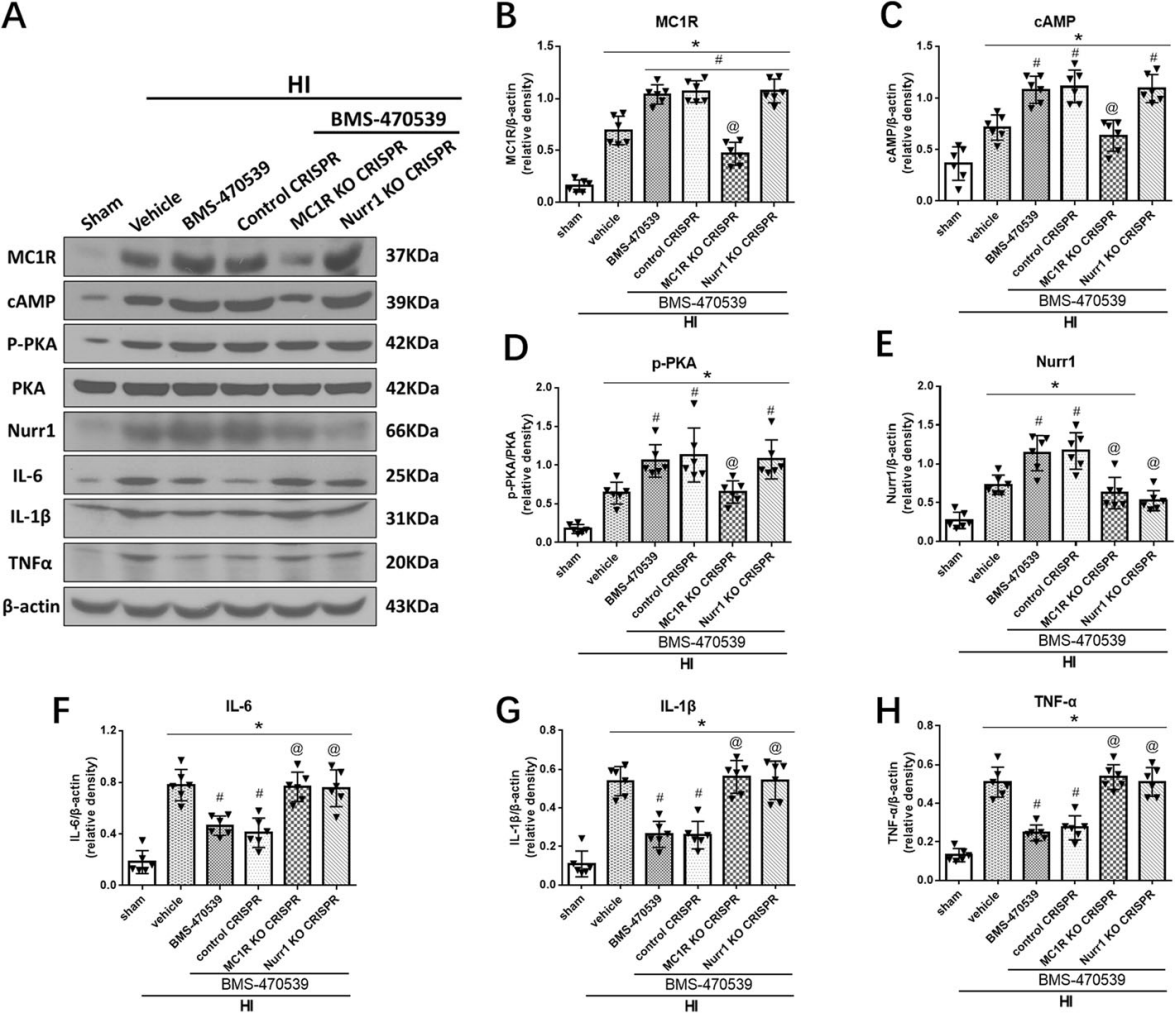
MC1R activation on neuroinflammation via cAMP/PKA/Nurr1 signaling pathway at 48 h post-HI. **a** Representative picture of Western blot data showing bands of the expression levels of MC1R, cAMP, p-PKA, Nurr1, IL-1β, TNFα, and IL-6 either with BMS-470539 treatment alone, BMS-470539 + MC1R KO CRISPR, BMS-470539 + Nurr1 KO CRISPR, and BMS-470539 + control CRISPR groups. **b**–**h** Western blot data quantification of bands showed that BMS-470539 treatment significantly increased the expression of MC1R, cAMP, p-PKA, and Nurr1 compared to the HI + vehicle group. Knockout of MC1R using CRISPR significantly decreased MC1R, cAMP, p-PKA, and Nurr1 expression levels compared to the HI + BMS-470539 group or HI + BMS-470539 + control CRISPR group. Furthermore, knockout of Nurr1 using CRISPR significantly decreased the levels of Nurr1, but did not affect MC1R, cAMP, and p-PKA expression compared to the corresponding controls. Activation of MC1R with BMS-470539 showed significantly decreased levels of IL-1β, TNFα, and IL-6, while both treatment groups with CRISPR interventions significantly reversed these effects. Data was represented as mean ± SD. The one-way ANOVA was followed by Tukey’s post hoc test (*p < 0.05 versus sham; ^#^*p* < 0.05 versus HI + vehicle; ^@^*p* < 0.05 HI + BMS-470539 or HI + BMS-470539 + control CRISPR; *n* = 6 per group)

To study the underlying mechanism through which MC1R activation attenuated neuroinflammation to exert its neuroprotective effects, the rat pups were randomly divided into the following group: sham, HI + vehicle (sterile saline), HI + BMS-470539, HI + BMS-470539 + MC1R KO CRISPR, HI + BMS-470539 + Nurr1 KO CRISPR, and HI + BMS-470539 + control CRISPR. Western blot was performed to evaluate the MC1R activation and its downstream signaling molecules at 48 h post-HI. The Western blot data showed that expression of MC1R, cAMP, p-PKA, Nurr1, IL-1β, TNFα, and IL-6 significantly increased in the HI + vehicle group compared to the sham group at 48 h post-HI (*p* < 0.05, Fig. 8a–h). Moreover, activation of MC1R with BMS-470539 further upregulated the expression of MC1R, cAMP, p-PKA, and Nurr1, while the expression of IL-1β, TNFα, and IL-6 were significantly decreased in the HI + BMS-470539 group compared to the HI + vehicle group (*p* < 0.05, Fig. 8a–h). However, knockout of MC1R with CRISPR significantly decreased the expression of MC1R, which abolished the effects of BMS-470539 (*p* < 0.05, Fig. 8a, b). Furthermore, knockout of MC1R with CRISPR significantly decreased the downstream protein expression including cAMP, p-PKA, and Nurr1 in the HI + BMS-470539 + MC1R KO CRISPR group compared to the HI + BMS-470539 group or HI + BMS-470539 + control CRISPR group (*p* < 0.05, Fig. 8a, c–e). Consistently, significant overexpression of IL-1β, TNFα, and IL-6 were observed in the HI + BMS-470539 + MC1R KO CRISPR group compared to the corresponding controls (*p* < 0.05, Fig. 8a, f–h).

Similarly, knockout of Nurr1 with CRISPR significantly decreased the levels of Nurr1, but increased the levels of IL-1β, TNFα, and IL-6 in the HI + BMS-470539 + Nurr1 KO CRISPR group compared to the HI + BMS-470539 group or HI + BMS-470539 + control CRISPR group. However, Nurr1 CRISPR did not change cAMP and p-PKA expression levels (*p* < 0.05, Fig. 8a, c–h).

## Discussion

Neonatal hypoxic-ischemic encephalopathy (HIE) is a devastating disease that results in life-long neurological disabilities, such as cerebral palsy, cognitive deficits, mental retardation, and epilepsy [2–4, 8]. HIE occurs in 1–8 per 1000 live births in developed countries, and as high as 26 per 1000 newborns in developing countries [1]. Current clinical therapies include supportive care and prevention of HIE complications [9–11]. However, safe and effective neuroprotective therapies for HIE are still lacking. Substantial studies revealed that neuroinflammation was a primary pathophysiological process after HI injury [5, 8, 12, 14, 15]. Hence, inhibition of neuroinflammation could be a safe and effective approach in reducing neurological impairment after HIE.

In the present study, we demonstrated that activation of melanocortin-1 receptor (MC1R) with BMS-470539 could attenuate neonatal HI-induced brain injury by suppressing neuroinflammation, which was partially attributed to the cAMP/PKA/Nurr1 signaling pathway. A summary of the novel findings in this study is as follows: Firstly, the expression levels of α-MSH increased over time, peaking at 24 h post-HI. The α-MSH showed colocalization with microglia, astrocytes, and neurons at 24 h post-HI. MC1R and Nurr1 expression levels increased post-HI, peaking at 48 h post-HI. MC1R and Nurr1 were expressed on microglia at 48 h post-HI. Secondly, activation of MC1R with BMS-470539 significantly reduced infarct area and brain atrophy, and improved short- and long-term neurological deficits post-HI. Thirdly, activation of MC1R with BMS-470539 promoted microglial polarization toward the M2 phenotype, and reduced peripheral immune cell infiltration at 48 h post-HI. Mechanistically, activation of MC1R with BMS-470539 increased the expression levels of MC1R, cAMP, p-PKA, and Nurr1, but decreased the expression of pro-inflammatory cytokines (TNFα, IL-6, and IL-1β) at 48 h post-HI. Finally, knockout of MC1R or Nurr1 by specific CRISPR reversed the neuroprotective effects of MC1R activation post-HI. Together, our findings demonstrated MC1R activation may serve as a potential therapeutic strategy to attenuate brain injury in infants with HIE.

It is known that neuroinflammation plays a critical role in mediating brain injury induced by HI [5, 9, 10, 15]. Previous studies showed that the activation of MC1R significantly attenuated brain injury by reducing neuroinflammation in SAH, ICH, and cerebral ischemia-reperfusion (I/R) injury [25, 26, 34]. However, the effect of MC1R in neonate HI is unknown. In this context, we hypothesized that activation of MC1R signaling pathway might exert an anti-inflammatory role post-HI. A previous study showed that the levels of MC1R significantly increased and reached a maximum point at 24 h after SAH in rats [25]. It has also been reported that the expression level of MC1R significantly increased and reached its peak at 72 h after ICH in mice [26]. Consistent with these studies, our time course results showed that MC1R expression levels significantly increased post-HI. However, the finding differed from the previous observations where it was shown that MC1R peaked at 24 h after SAH in rats and 72 h after ICH in mice, whereas our time course results showed that MC1R expression levels peaked at 48 h post-HI. We suppose that such discrepancy may be due to the differing mechanisms of injury across these animal models. In the present study, we found that MC1R were colocalized with microglia at 48 h post-HI. The finding is consistent with previous studies where MC1R was primarily expressed on microglia [25, 26]. To date, five melanocortin receptors (MCRs) have been identified and termed MC1R to MC5R, among which MC1R, MC3R, and MC4R have been reported to be expressed in the central nervous system [55, 56]. MC2R is expressed in the adrenal gland and adipocytes, and MC5R is expressed in peripheral tissues [24, 57]. MC3R and MC4R have been shown to exert anti-inflammatory effects [34, 58]. Since MC3R and MC4R may also be influenced by HI, we did Western blot to measure their expression. Our results showed that MC3R and MC4R increased at 48 h post-HI. In the present study, we focused on MC1R and its downstream signaling pathway in neuroinflammation. Therefore, to demonstrate that MC1R, and not other MCRs, play anti-inflammatory and neuroprotective effects in HI brain injury, a specific selective agonist of MC1R, BMS-470539, was used in our study. Furthermore, a specific MC1R CRISPR KO plasmid was also used.

MC1R is activated by its agonist α-melanocyte-stimulating hormone (α-MSH) [30, 59]. The α-MSH had been demonstrated to have the highest binding affinity for MC1R [55, 56, 58]. In our study, we were able to demonstrate that α-MSH was expressed on microglia, neurons, and astrocytes. And α-MSH showed higher expression in neurons compared to microglia and astrocytes. Our time course results showed that the expression levels of α-MSH increased over time and peaked at 24 h post-HI. In the present study, the Western blot results showed that the expression of α-MSH significantly increased in the ipsilateral hemisphere compared to the sham group and the contralateral hemisphere at 24 h post-HI. However, there is no significant difference between the sham group and the contralateral hemisphere. In a recent study, immunofluorescence staining showed that there was no significant difference in protein expression between the sham group and the contralateral hemisphere after HI [52]. In a rat model of HI brain injury, only the ipsilateral hemisphere was used to measure the protein expression by Western blot, as previously reported [5, 6, 46]. Based on the above results, we used the ipsilateral hemisphere tissues to perform quantitative protein analysis using Western blot in a rat neonatal HI model. There is no doubt that both the protective and harmful factors would increase after an injury as the body attempts to maintain homeostasis. The α-MSH functions as a protective factor by reducing inflammation, apoptosis, oxidative stress, etc. [26, 59]. This finding can be explained by the upregulation of α-MSH gene expression in brain cells (microglia, neurons, astrocytes, etc.) post-HI. In the present study, the results showed that α-MSH and MC1R expression levels increased post-HI. Moreover, previous studies showed that treatment with α-MSH upregulated the expression of MC1R [27, 60, 61]. We assume that there might be a correlation between MC1R and α-MSH, and that α-MSH may be one of the main reasons for increasing MC1R post-HI. In the present study, our TTC staining images showed that much of the ipsilateral hippocampus and striatum seemed to be protected in the BMS-470539 treatment group. When the right common carotid artery is severed in a rat model of HI brain injury, the blood flow to the cortex tissue is almost completely blocked that causes irreversible injury and subsequent cell death; however, the ipsilateral hippocampus and striatum have only partially reduced blood flow due to the circle of Willis. In those regions, tissues have the potential for recovery in the early stage [62, 63]. Moreover, previous studies have shown that MC1R and α-MSH are expressed in the central nervous system including striatum and hippocampus [64–66]. Activation of MC1R with BMS-470539 has been demonstrated to exert anti-inflammatory and neuroprotective effects in our study. These are major reasons why the striatum and hippocampus seem to be protected in the BMS-470539 treatment group in particular.

Nurr1 reportedly provides significant protection against inflammation by inhibiting expression of pro-inflammatory cytokines in microglia and astrocytes, whereas a reduction in Nurr1 expression exaggerates the inflammatory responses [35, 67, 68]. Moreover, Nurr1 activation reportedly promotes microglial polarization toward the M2 phenotype [69, 70]. Activation of Nurr1 with the agonist, amodiaquine, attenuated inflammatory events and neurological deficits in a mouse model of ICH [37]. In a rat model of acute cerebral ischemic/reperfusion, overexpression of Nurr1 inhibited TNFα expression on microglia, and attenuated the ischemia/reperfusion-induced inflammatory response [36]. Previous studies found that activation of Nurr1 using agonists provided important neuroprotective effects against neuroinflammation in Parkinson’s disease, Alzheimer’s disease, and multiple sclerosis [67, 71–73]. It has also been reported that inflammatory stimuli, such as lipopolysaccharides (LPS), upregulated Nurr1 expression in cultured mouse microglia [74]. Previous studies showed that Nurr1 activation attenuated inflammation, and Nurr1 knockout induced early experimental autoimmune encephalomyelitis (EAE) onset and increased inflammatory infiltration [75, 76]. On the contrary, Doi and colleagues showed that Nurr1 has a pro-inflammatory effect by increasing the expression of IL-17 and IFN-γ in CD4^+^ T cells of EAE [77]. Moreover, Trudler and coworkers reported that Nurr1 was expressed in CD4^+^T cells and had pro-inflammatory effects by increasing the expression of IL-2 and IL-17 in splenocytes and CD4^+^T cells from EAE mice [78]. The role of Nurr1 in CD4^+^T cells from EAE mice was controversial. However, there was no data that show that Nurr1 exerts pro-inflammatory effects in microglia and astrocytes. The reported scientific literature suggested that Nurr1 was a promising therapeutic target for neuroprotective therapy against neuroinflammation [35]. In the present study, our time course results showed that the expression levels of Nurr1 were increased in parallel with MC1R post-HI. Therefore, the above data indicates that Nurr1 might participate in MC1R-mediated anti-inflammatory effects. Nurr1 has been shown to be expressed on microglia [67, 74, 79]. Consistent with these studies, our results showed that Nurr1 was colocalized with microglia at 48 h post-HI.

BMS-470539, a novel potent and specific selective agonist of MC1R, reportedly exerts anti-inflammatory effects in in vivo and in vitro experiments [25, 31–34]. In a mice model of lung inflammation, BMS-470539 reduced inflammation by activating MC1R [32]. BMS-470539 reportedly displays anti-inflammatory and chondroprotective effects in the human chondrocyte cell-line [31]. Activation of MC1R with BMS-470539 has also been demonstrated to exert anti-inflammatory and neuroprotective effects in a rat model of SAH [25]. There are some limitations to α-MSH as an anti-inflammatory target, such as a lack of selectivity and possible involvement in lipid metabolism [32, 80]. Furthermore, Schulte-Herbrüggen and coworkers reported that α-MSH could promote spontaneous post-ischemic pneumonia by inhibiting pulmonary antibacterial defenses [81]. For the above reasons, we chose BMS-470539 as the treatment for activating MC1R in this study. The neuroinflammatory response is characterized by activation of microglia and migration of peripheral macrophages in acute HI brain injury [15]. Resident microglia were activated rapidly within minutes after cerebral ischemia including HI injury [82, 83]. Blood-derived macrophages were recruited into the ischemic brain tissue with a delay of hours to a few days [84, 85]. The majority of macrophages in the infarct area were derived from resident microglia, preceding and predominating over blood-derived macrophages [86, 87]. Previous studies have shown that resident microglia, rather than peripherally derived macrophages, plays more major contributions in acute HI brain injury [88, 89]. M1 microglia release pro-inflammatory cytokines (TNFα, IL-6, and IL-1β) and exacerbate neuronal injury, whereas the M2 phenotype of microglia promotes anti-inflammatory responses that are reparative and neuroprotective [13, 90–92]. In the present study, the results showed that activation of MC1R with BMS-470539 increased the expression of CD206 and reduced the expression of IL-1β and MPO. However, knockout of MC1R or Nurr1 with CRISPR significantly reversed these effects. Based on the above evidence, we confirmed that activation of MC1R with BMS-470539 downregulated pro-inflammatory cytokines, promoted microglial M2 polarization, and reduced peripheral immune cell infiltration.

To examine whether activation of MC1R with BMS-470539 may have neuroprotective roles, we investigated the influence of BMS-470539 treatment on brain damage and neurological functions at 48 h and 28 days post-HI. We evaluated the optimal dose of BMS-470539, determined by the infarct area, weight loss, and neurological function at 48 h post-HI. Three doses of BMS-470539 were delivered intranasally at 1 h post-HI, low (50 μg/kg), medium (160 μg/kg), and high (500 μg/kg). Both medium and high doses of BMS-470539 groups significantly reduced infarct area and body weight loss, and improved short-term neurological function at 48 h post-HI. However, there is no significant difference between these groups. Based on the above results and the principle of priority, we chose the medium dose of BMS-470539 for long-term outcome and mechanism studies. In this study, knockout of MC1R or Nurr1 with CRISPR significantly reversed the neuroprotective effects of MC1R activation by increasing infarct area and body weight loss, impairing short-term neurological function at 48 h post-HI. Moreover, MC1R activation with BMS-470539 reduced brain atrophy and improved long-term neurological function at 28 days post-HI. Similarly, MC1R activation with BMS-470539 improved rats’ performance in short- and long-term tests after SAH, as seen from previous study [25].

We further explored the molecular basis of MC1R-mediated anti-inflammatory effects post-HI. MC1R is coupled to adenylyl cyclase and mediates its effects primarily by activating a cAMP-dependent signaling pathway [24, 57]. Activation of MC1R stimulated cAMP production and enhanced DNA repair after ultraviolet irradiation [30]. The MC1R-cAMP pathway plays a role in attenuating ultraviolet-induced oxidative stress via activation of p53 [93]. Intriguingly, an elevation of intracellular cAMP levels can also increase PKA’s phosphorylation [42–44]. Coincidentally, PKA has been established as an upstream regulatory molecule of Nurr1, as is seen in previous studies [38–40]. The common pathway of Nurr1-related inflammation and cancer was the cAMP/PKA signaling pathway [40, 41]. In our study, activation of MC1R with BMS-470539 further upregulated the expression of MC1R, cAMP, p-PKA, and Nurr1, but downregulated the expression of pro-inflammatory cytokines (TNFα, IL-6, and IL-1β). Based on the above results, we speculate that MC1R activation with BMS-470539 may increase the level and activity of MC1R. Knockout of MC1R with CRISPR abolished the neuroprotective effects of MC1R activation by decreasing MC1R expression level and its downstream molecules, cAMP, p-PKA, and Nurr1, and increasing the expressions of TNFα, IL-6, and IL-1β. Moreover, knockout of Nurr1 with CRISPR had no effects on the expression of cAMP and PKA’s phosphorylation, while it significantly decreased the levels of Nurr1 and increased the levels of IL-1β, TNFα, and IL-6. Thus, our data suggested that activation of MC1R with BMS-470539 upregulated the Nurr1 expression, at least in part, via the cAMP/PKA signaling pathway, leading to the anti-inflammatory effects post-HI.

There are several limitations to our study. Firstly, activation of MC1R may exert its anti-inflammatory effects through other signaling pathways [25, 26]. Hence, future studies are needed to evaluate other potential mechanisms of action after HIE. Secondly, both previous studies and our study showed that the MC1R was expressed on astrocytes and neurons [25]. Hence, the anti-apoptotic effects and anti-oxidative stress protection of MC1R activation post-HI require further investigation. Thirdly, in our study, we observed an increase in α-MSH and MC1R post-HI. However, the mechanism behind the upregulation of α-MSH and MC1R after HI injury is poorly understood. Therefore, future studies are necessary to focus on those possible underlying mechanisms.

**Fig. 9:**
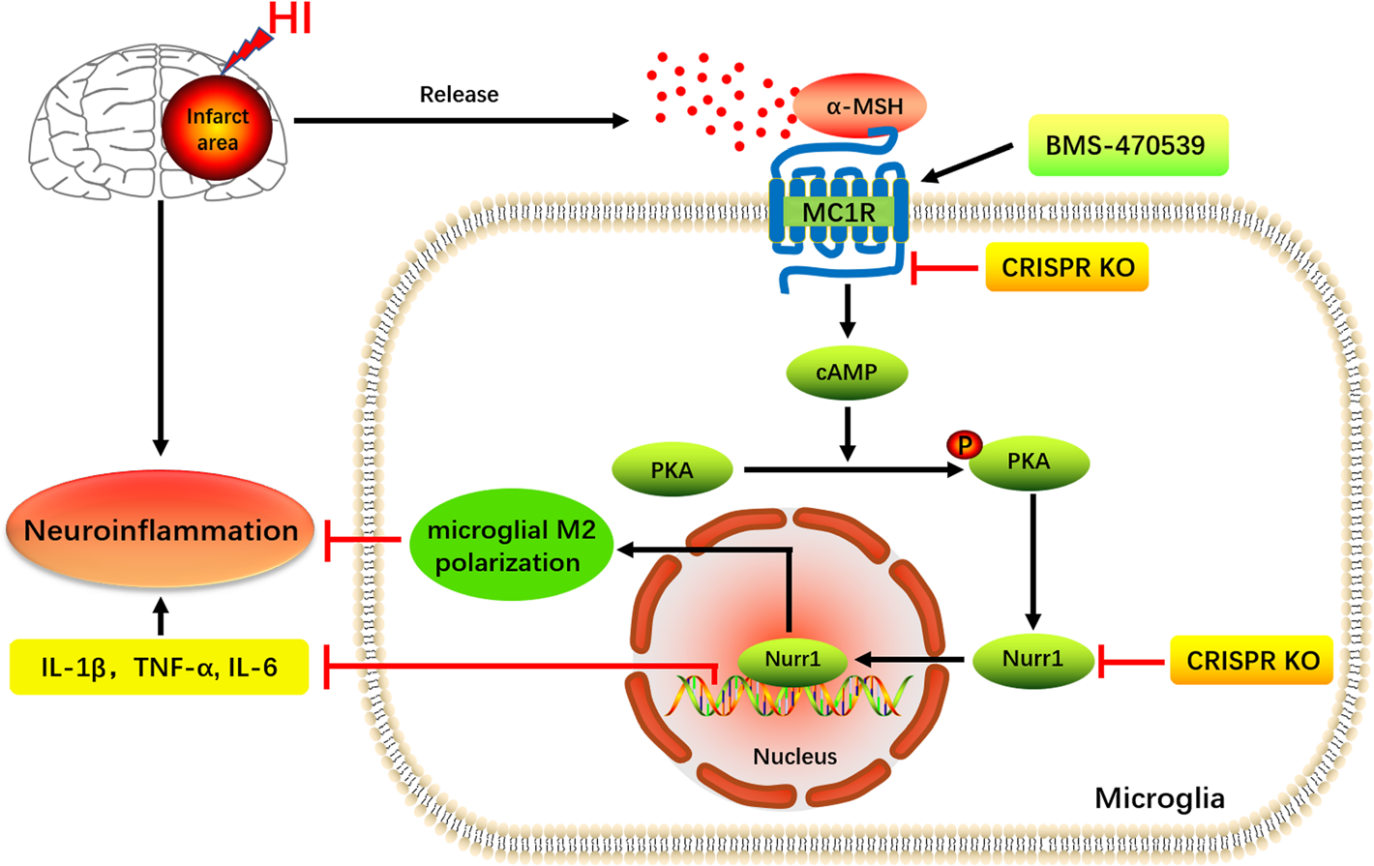
The potential molecular mechanisms of anti-inflammatory and neuroprotective effects through the MC1R/cAMP/PKA/Nurr1 signaling pathway post-HI

In conclusion, activation of MC1R with BMS-470539 downregulated pro-inflammatory cytokines, promoted microglial M2 polarization, reduced peripheral immune cell infiltration, and improved neurological deficits after neonatal HI brain injury in rats. Such anti-inflammatory and neuroprotective effects were mediated, at least in part, via the cAMP/PKA/Nurr1 signaling pathway (Fig. 9). Therefore, MC1R activation might be a promising therapeutic target for infants with HIE.

## Supplementary Information

**Additional file 1: Figure S1.** Expression of α-MSH in the contralateral hemisphere and the ipsilateral hemisphere at 24 h post-HI. (A) Representative pictures of Western blot data. (B)Western blot data showed that the expression level of α-MSH significantly increased in the ipsilateral hemisphere at 24 h post-HI, and no significant difference between the sham group and the contralateral hemisphere. Data were represented as mean ± SD. Statistical differences between groups were analyzed using one-way ANOVA, followed by Tukey's post-hoc test. (*p<0.05 versus sham, #*p*<0.05 versus contralateral hemisphere; n=4 per group). **Figure S2.** Expression levels of MC3R and MC4R at 48h post-HI. (A) Representative pictures of Western blot data. (B-C) Western blot data showed that the expression of MC3R and MC4R significantly increased after HI. Data were represented as mean ± SD. Statistical differences between groups were analyzed using Student's t test, followed by Student–Newman–Keuls test. (*p<0.05 versus sham; n=4 per group). **Figure S3.** Quantification of α-MSH-positive cells. α-MSH showed higher expression in neurons compared to microglia and astrocytes. Data were represented as mean ± SD. Statistical differences between groups were analyzed using one-way ANOVA, followed by Tukey's post-hoc test. (*p<0.05 versus Iba-1(+), #*p*<0.05 versus GFAP (+); n=2 per group).

## Data Availability

The datasets analyzed during the current study are available from the corresponding author on reasonable request.

## Abbreviations

MC1R: Melanocortin-1 receptor
α-MSH: α-melanocyte-stimulating hormone
cAMP: Cyclic adenosine monophosphate
PKA: Protein kinase A
Nurr1: Nuclear receptor related factor 1
KO: Knockout
HI: Hypoxic-ischemic
HIE: Hypoxic-ischemic encephalopathy
SAH: Subarachnoid hemorrhage
ICH: Intracerebral hemorrhage
EAE: Experimental autoimmune encephalomyelitis
GFAP: Glial fibrillary acidic protein
Iba-1: Ionized calcium-binding adaptor molecule 1
NeuN: Neuronal specific nuclear protein
PBS: Phosphate-buffered saline
TTC: 2,3,5-Triphenyltetrazolium chloride monohydrate
ARRIVE: Animal Research: Reporting of In Vivo Experiments
