# Supplementary material for: Activation of MC1R with BMS-470539 attenuates neuroinflammation via cAMP/PKA/Nurr1 pathway after neonatal hypoxic-ischemic brain injury in rats
Source: J Neuroinflammation. 2021 Jan 19;18:26. doi: 10.1186/s12974-021-02078-2 (PMC7814630; doi:10.1186/s12974-021-02078-2)
Supplement: Supplementary file 1 — Additional file 1: Figure S1. Expression of α-MSH in the contralateral hemisphere and the ipsilateral hemisphere at 24 h post-HI. (A) Representative pictures of Western blot data. (B)Western blot data showed that the expression level of α-MSH significantly increased in the ipsilateral hemisphere at 24 h post-HI, and no significant difference between the sham group and the contralateral hemisphere. Data were represented as mean ± SD. Statistical differences between groups were analyzed using one-way ANOVA, followed by Tukey's post-hoc test. (*p<0.05 versus sham, #p<0.05 versus contralateral hemisphere; n=4 per group). Figure S2. Expression levels of MC3R and MC4R at 48h post-HI. (A) Representative pictures of Western blot data. (B-C) Western blot data showed that the expression of MC3R and MC4R significantly increased after HI. Data were represented as mean ± SD. Statistical differences between groups were analyzed using Student's t test, followed by Student–Newman–Keuls test. (*p<0.05 versus sham; n=4 per group). Figure S3. Quantification of α-MSH-positive cells. α-MSH showed higher expression in neurons compared to microglia and astrocytes. Data were represented as mean ± SD. Statistical differences between groups were analyzed using one-way ANOVA, followed by Tukey's post-hoc test. (*p<0.05 versus Iba-1(+), #p<0.05 versus GFAP (+); n=2 per group). [file 12974_2021_2078_MOESM1_ESM.pdf]

Activation of MC1R with BMS-470539 attenuates neuroinflammation via cAMP/PKA/Nurr1 pathway after neonatal hypoxic–ischemic brain injury in rats

Shufeng Yu[1,2], Desislava Met Doycheva[2], Marcin Gamdzyk[2], Yijun Yang[3], Cameron Lenahan[2,4], Gaigai Li[2,5], Dujuan Li[2,6], Lifei Lian[2,5], Jiping Tang[2], Jun Lu[1*], and John H. Zhang[2, 7*].

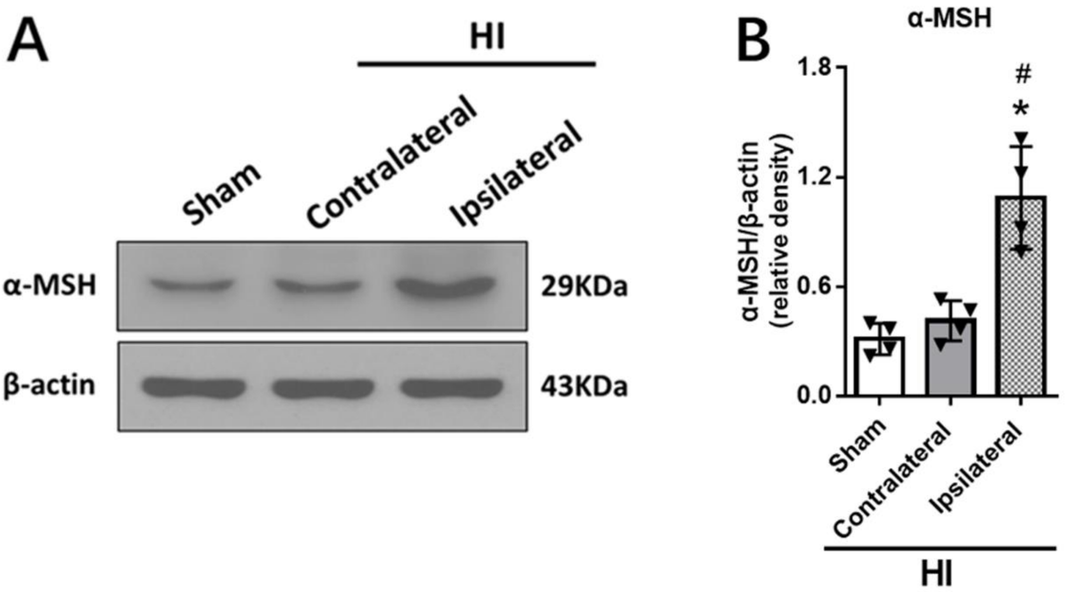

Supplementary Figure 1. Expression of α-MSH in the contralateral hemisphere and the ipsilateral hemisphere at 24h post HI. (A) Representative pictures of Western blot data. (B)Western blot data showed that the expression level of α-MSH significantly increased in the ipsilateral hemisphere at 24 h post HI, and no significant difference between the sham group and the contralateral hemisphere. Data were represented as mean ± SD. Statistical differences between groups were analyzed using one-way ANOVA, followed by Tukey's post-hoc test. (**p*<0.05 versus sham, #*p*<0.05 versus contralateral hemisphere; n=4 per group).

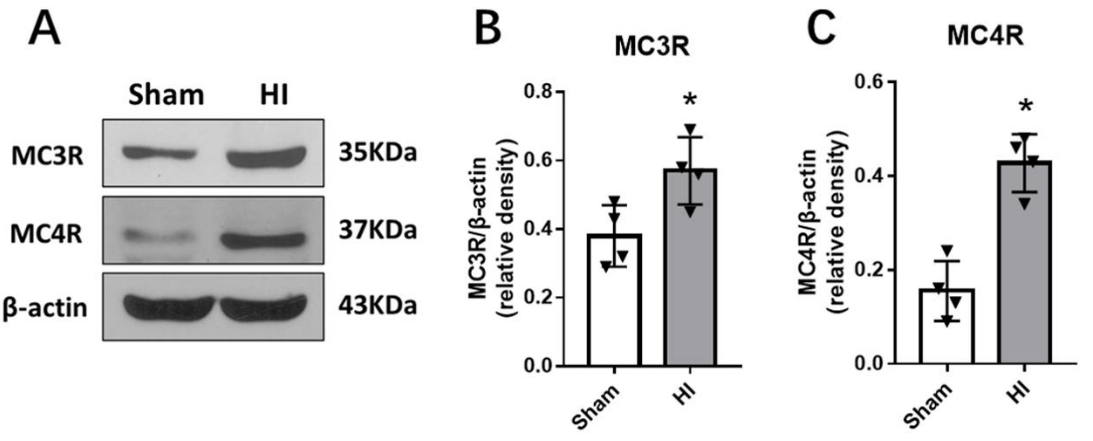

Supplementary Figure 2. Expression levels of MC3R and MC4R at 48h post-HI. (A) Representative pictures of Western blot data. (B-C) Western blot data showed that the expression of MC3R and MC4R significantly increased after HI. Data were represented as mean ± SD. Statistical differences between groups were analyzed using Student's *t* test, followed by Student-Newman-Keuls test. (*$p<0.05$ versus sham; n=4 per group).

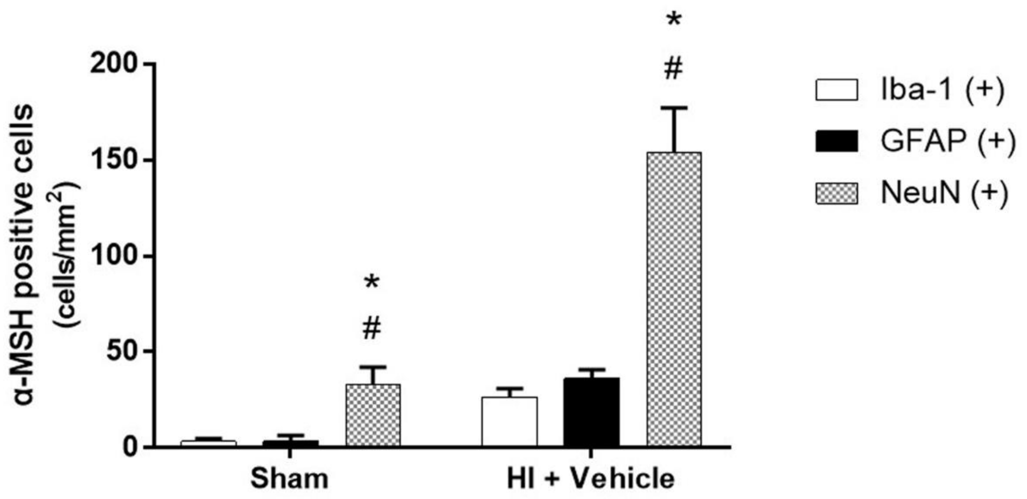

Supplementary Figure 3. Quantification of α-MSH-positive cells. α-MSH showed higher expression in neurons compared to microglia and astrocytes. Data were represented as mean ± SD. Statistical differences between groups were analyzed using one-way ANOVA, followed by Tukey's post-hoc test. (*$p<0.05$ versus Iba-1(+), #$p<0.05$ versus GFAP (+); n=2 per group).
